# Physiological Responses of Dominant Alpine Plant Species to Environmental Gradients on the Tibetan Plateau

**DOI:** 10.3390/plants15050719

**Published:** 2026-02-27

**Authors:** Xiaotong Liu, Junxi Wu, Huanyu Zhou, Xianlei Gao, Lanlan Ye, Xiaofang Huang, Xianzhou Zhang, Mingxue Xiang, Ying Pan

**Affiliations:** 1 Lhasa Plateau Ecosystem Research Station, Key Laboratory of Ecosystem Network Observation and Modelling, Institute of Geographic Sciences and Natural Resources Research, Chinese Academy of Sciences, Beijing 100101, China; liuxiaotong5027@igsnrr.ac.cn (X.L.); zhouhuanyu2419@igsnrr.ac.cn (H.Z.); yell02200059@163.com (L.Y.); huangxiaofang5960@igsnrr.ac.cn (X.H.); zhangxz@igsnrr.ac.cn (X.Z.); 2 College of Resources and Environment, University of Chinese Academy of Sciences, Beijing 100190, China; 3 School of Ecology and Environment, Tibet University, Lhasa 850000, China; g18221991182@163.com; 4 State Key Laboratory of Plateau Ecology and Agriculture, Qinghai University, Xining 810018, China; xiangmx.20b@igsnrr.ac.cn

**Keywords:** physiological traits, Tibetan Plateau, temperature and precipitation, alpine plants, stress

## Abstract

Understanding how plant physiological traits respond to environmental variation is essential for explaining plant performance in alpine ecosystems. Based on field sampling along an elevational transect on the Tibetan Plateau, we quantified osmotic adjustment compounds, antioxidant indicators, and plant hormones in leaves of different species to examine interspecific differences in sensitivity to temperature and precipitation to characterize patterns of physiological plasticity among alpine plants. Along the elevational gradient, declining temperature results in increasing cold stress, whereas lower elevations are associated with reduced precipitation and intensified drought stress. Temperature primarily influenced plant physiological trait expression by promoting growth-related physiological processes, while precipitation variability mainly regulated traits associated with water stress. The three dominant alpine meadow species exhibited distinct patterns of physiological plasticity: *Poa litwinowiana* showed coordinated regulation of growth and defense pathways, whereas *Carex moorcroftii* and *Carex parvula* displayed more conservative response strategies, with physiological regulation tending to maintain homeostasis rather than strongly activating stress responses. These interspecific differences in physiological regulation were significantly associated with variations in plant height, cover, and dominance, providing trait-level physiological insights relevant to plant performance.

## 1. Introduction

Altitudinal gradients represent a crucial environmental factor influencing plant growth, morphology, and physiology [1,2]. These gradients serve as powerful “natural experiments” for assessing the ecological and evolutionary responses of biomes to geophysical drivers [3]. In alpine regions, altitudinal gradients induce dramatic environmental shifts over relatively short spatial distances, notably in temperature, humidity, and atmospheric pressure [4]. In general, increasing elevation is associated with decreasing temperature, carbon dioxide, and oxygen concentrations, while precipitation and light intensity typically increase [5]. Such environmental modifications drive adaptive changes in plant morphology and physiology, enabling species to withstand the unique stresses imposed by elevation [6,7]. Furthermore, ecophysiological traits play a critical role in determining how plant species respond to environmental changes [4,8,9].

Changes in temperature and moisture with increasing altitude constitute a key environmental factor influencing plant survival, distribution, growth, and reproduction [10]. To cope with the extreme cold conditions at high elevations, plants have evolved specialized cold-tolerance mechanisms [11,12]. Consequently, plant species in alpine and other cold-climate regions often exhibit conservative life-history strategies, including slow growth rates, small stature, reduced and toughened leaf structures, lower specific leaf area (SLA), and higher leaf dry matter content [13,14]. At the physiological level, plants at high altitudes undergo extensive metabolic adjustments in response to low temperatures. Cold stress triggers modifications in multiple metabolic pathways, including the accumulation of osmolytes and the enhancement of antioxidant defense systems to mitigate oxidative stress [15,16,17]. Sucrose accumulation and protein synthesis are commonly observed in cold-tolerant plant species during cold stress and are generally positively correlated with cold tolerance [18,19]. Experimental studies have demonstrated that altitude exerts a significant influence on proline and malondialdehyde (MDA) accumulation, as well as on antioxidant enzyme activities in plant leaves [20,21]. Plant endogenous hormones, including gibberellins (GA), salicylic acid (SA), and indole-3-acetic acid (IAA), play essential roles in regulating plant water balance, osmotic stress responses, and adaptive mechanisms to abiotic stress, thereby influencing growth and developmental processes [22,23,24,25,26]. Additionally, elevation-induced environmental changes have also been shown to affect the concentrations of abscisic acid (ABA), indole-3-acetic acid (IAA), and gibberellic acid in plant leaves [27]. For example, higher accumulation of endogenous plant hormones (ABA) is commonly associated with response to environmental stresses such as low temperature [28]. Therefore, the study of plant and physiological attributes in response to altitudinal gradients is a powerful tool for understanding how plant communities behave under different environmental stresses.

Along altitudinal gradients, plants cope with environmental variation through coordinated physiological regulation that supports growth maintenance and stress tolerance, which in turn shapes life-history strategies and performance trade-offs under complex conditions. Despite substantial progress in understanding plant responses to altitudinal gradients in various mountain systems worldwide [29,30,31,32], relatively few studies have focused on the integrated physiological mechanisms that enable alpine plants to cope with the combined cold and arid stresses characteristic of high-altitude environments on the Tibetan Plateau [33]. Previous studies along altitudinal gradients have provided valuable insights into growth patterns and individual physiological traits of shrubs and herbaceous species [34,35]. However, these studies have largely emphasized specific growth parameters or isolated trait variations—such as nutritional composition, reproductive traits, photosynthetic performance, and functional leaf traits [13,33], rather than examining comprehensive, multi-pathway physiological responses to altitudinal variation. As a result, empirical evidence linking integrated physiological regulation to patterns of plant performance and relative dominance under rapidly changing high-altitude environmental conditions remains limited, particularly in the Tibetan Plateau region.

The fragile alpine ecosystem of the Tibetan Plateau is highly sensitive to climate change and anthropogenic disturbances, making it particularly vulnerable to the effects of global warming and human-induced environmental changes [36,37,38]. Among the characteristic plant species of this region, *Carex parvula* (*Cyperaceae*) and *Carex moorcroftii* (*Cyperaceae*) are perennial herbaceous plants endemic to the Tibetan Plateau, serving as key foundation species in alpine meadows [39,40]. Additionally, *Poa litwinowiana* (*Poaceae*) is widely distributed across Central and South Asia and represents one of the dominant species in alpine grasslands [41]. These three species occur across a broad altitudinal range (2500–5000 m), indicating strong adaptability to alpine climatic conditions. Their prevalence in alpine communities highlights their ecological importance in vegetation composition and environmental adaptation. Accordingly, this study aimed to examine physiological processes related to osmotic adjustment, oxidative stress defense, and hormonal regulation in three dominant alpine grasses along an altitudinal gradient. It further assessed how variation in these physiological traits is associated with patterns of plant performance across elevations.

Based on the above research gaps, this study hypothesized that (a) physiological traits of alpine plants vary systematically along the altitudinal gradient in association with altitude-related environmental factors; (b) alpine plant species exhibit distinct physiological responses to altitudinal variation, resulting in contrasting sensitivities to environmental stress.

## 2. Results

### 2.1. Physiological Trait Differentiation in Plants Along Altitudinal Gradients

**Figure 1:**
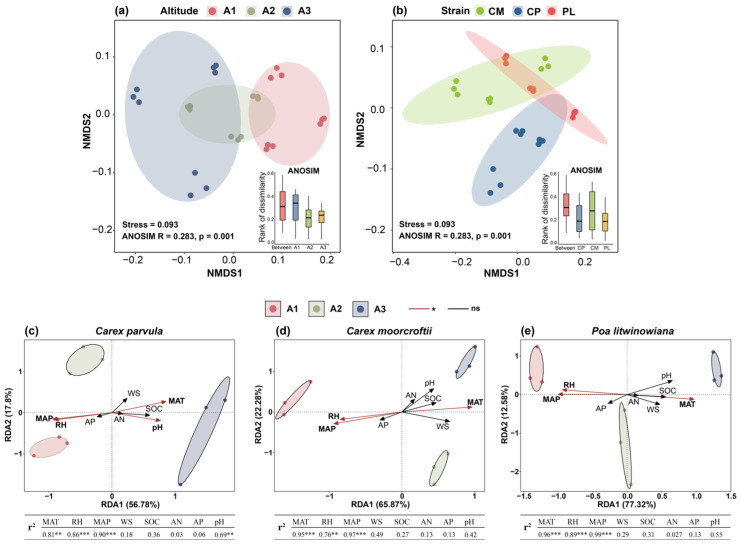
Altitude-and species-specific variation in plant physiological responses and their environmental associations. (**a**) NMDS plot showing differences in physiological trait composition among altitude groups (A1: 4000–4100 m, A2: 4500–4600 m, A3: 5000–5100 m). Colored ellipses represent 95% confidence areas. (**b**) NMDS plot showing physiological trait composition differences across three plant species or strains (CP: *Carex parvula*, CM: *Carex moorcroftii*, and PL: *Poa litwinowiana*). Both A and B are based on Bray–Curtis dissimilarity using scaled physiological indicators. ANOSIM results (R and *p* values) indicate significant group separation. (**c**) Redundancy analysis (RDA) of *Carex parvula* relating physiological traits to environmental and soil factors. (**d**) Redundancy analysis of *Carex moorcroftii* relating physiological traits to environmental and soil factors. (**e**) Redundancy analysis of *Poa litwinowiana* relating physiological traits to environmental and soil factors. Arrow length indicates the strength of correlation; red arrows denote significant predictors (*p* < 0.05). Sample proximity to arrows reflects the strength and direction of influence from each factor. Significance levels of predictors are shown as: *p* < 0.05 (*), *p* < 0.01 (**), and *p* < 0.001 (***); “ns” denotes non-significance.

To explore how alpine dominant species physiologically respond to cold and drought stress, we first analyzed overall differences in physiological trait concentrations along an altitudinal gradient. Non-metric multidimensional scaling (NMDS) showed that samples of the three dominant plants exhibited significant separation in elevation and species (Stress = 0.093, ANOSIM R = 0.283, *p* = 0.001), suggesting that physiological traits are significantly structured across environmental gradients (Figure 1a,b). Redundancy analysis (RDA) further showed that the climatic factors, especially mean annual temperature (MAT) and mean annual precipitation (MAP), together explained up to 89.9% of the variation in physiological traits (RDA1 = 77.32%, RDA2 = 12.59%), while soil factors (e.g., SOC, pH) contributed less (Figure 1c–e). The RDA model explained more than 70% of the differences in physiological characteristics of different species, indicating a close relationship between climatic factors and plant physiological processes.

### 2.2. Variation in Structural Traits and Physiological Responses of Dominant Alpine Plants

Plant diversity increased with decreasing elevation, with the number of species increasing from 18 to 35, accompanied by a decrease in total cover, suggesting that the warmer, drier conditions may have provided ecological niche space and habitat heterogeneity for more plant species (Table A1). The three dominant plant species showed different changes in community establishment capacity in response to changes in precipitation and temperature (Table 1): *Carex parvula* showed high cover and density; *Carex moorcroftii* was stable in all indicators of establishment; and *Poa litwinowiana*, despite its high individual heights, was weakly established, with only slight enhancement in warmer areas.

**Figure 2:**
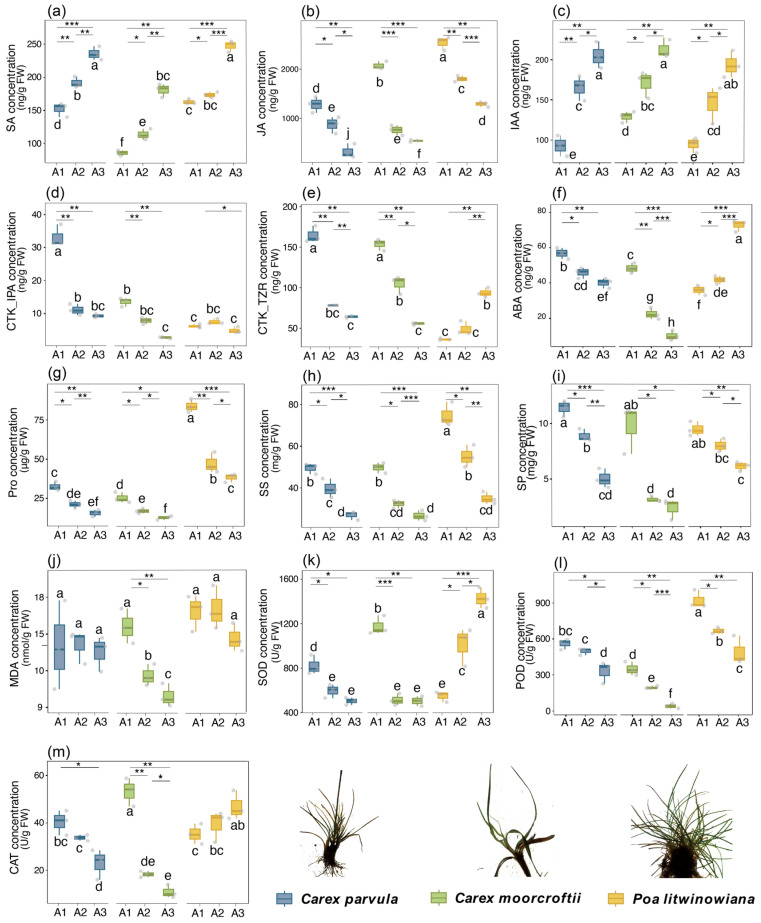
Ecophysiological trait indicators of *Carex parvula*, *Carex moorcroftii*, and *Poa litwinowiana* across an altitudinal gradient, based on various physiological variables. Different letters are shown as species means and least significant differences (LSD) across altitudes. A single asterisk (*) indicates significance at *p* < 0.05, double asterisks (**) at *p* < 0.01, and triple asterisks (***) at *p* < 0.001. (**a**–**m**) SA, JA, IAA, CTK_IPA, CTK_TZR, ABA, Pro, SS, SP, MDA, SOD, POD, and CAT, respectively.

Plant physiological indicators reflected the metabolic regulation trends and environmental adaptation mechanisms of the species in the context of warm-drying, with significant differences in hormone levels, osmoregulation capacity and antioxidant defense systems (Figure 2). Overall, most of the physiological indicators of the three species tended to increase or decrease in parallel with the increase in the degree of warming and drying, indicating broad shifts in metabolic activity associated with environmental variation. *Poa litwinowiana* exhibited higher concentrations of most physiological indicators under warmer and drier conditions, implying enhanced activity in hormone regulation, osmotic adjustment, and reactive oxygen species (ROS) scavenging pathways in response to stress. In contrast, *Carex moorcroftii* showed a general decline in physiological trait levels under similar conditions. *Carex parvula* displayed intermediate values across most indicators, with relatively small fluctuations, indicating a degree of physiological buffering capacity under stress.

Notably, the three species exhibited distinct patterns in key drought-responsive physiological traits, including ABA, the cytokinin trans-zeatin riboside (CTK_TZR), and the antioxidant enzyme POD (Figure 2). In *Poa litwinowiana*, concentrations of these indicators increased with intensifying warming and drying, whereas *Carex parvula* and *Carex moorcroftii* showed overall declines. This contrasting trend suggests that *Poa litwinowiana* exhibits stronger upregulation of stress-related physiological traits under arid conditions while maintaining relatively higher levels of growth-associated hormonal signals, reflecting a physiological response profile that balances defense activation with continued growth.

### 2.3. Mechanistic Differences in the Dominant Regulatory Roles of Temperature and Precipitation

**Figure 3:**
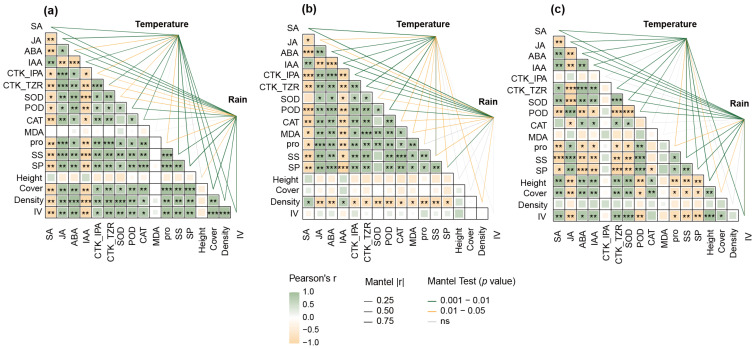
Correlation and Climatic Response Structures of Physiological and Structural Traits in Three Alpine Plant Species. (**a**) *Carex parvula*, (**b**) *Carex moorcroftii*, and (**c**) *Poa litwinowiana*. Green and orange lines indicate significant Mantel correlations with temperature and precipitation, respectively. Pearson’s r values are color-coded, with asterisks indicating levels of significance (* *p* < 0.05, ** *p* < 0.01, *** *p* < 0.001.).

To investigate the associations between plant physiological traits, structural attributes, and meteorological variables, we constructed a composite correlation framework based on Spearman correlation and Mantel tests. The Mantel test results showed that physiological traits and dominance-related structural attributes of the three species exhibited varying degrees of association with temperature and precipitation gradients. Most of the physiological indicators, including peroxidase activity (POD), proline (PRO), soluble sugars (SS), and soluble proteins (SP), were significantly positively correlated with each other. were significantly positively correlated with each other, and some degree of correlation also existed among hormonal indicators, especially between ABA and CTK_TZR, which showed strong positive correlation among the three plants. *Poa litwinowiana* (Figure 3c) showed the strongest structural correlation with meteorological variables, indicating that it was the most sensitive to temperature changes; *Carex moorcroftii* (Figure 3b) showed a weaker relationship with environmental gradients at the level of population indicators, showing a certain degree of conservatism in environmental adaptations; *Carex parvula* (Figure 3a) showed highly significant structural correlations with meteorological variables in several key physiological indicators, reflecting that it had stronger integration and regulation of its physiological pathways along environmental gradients.

**Figure 4:**
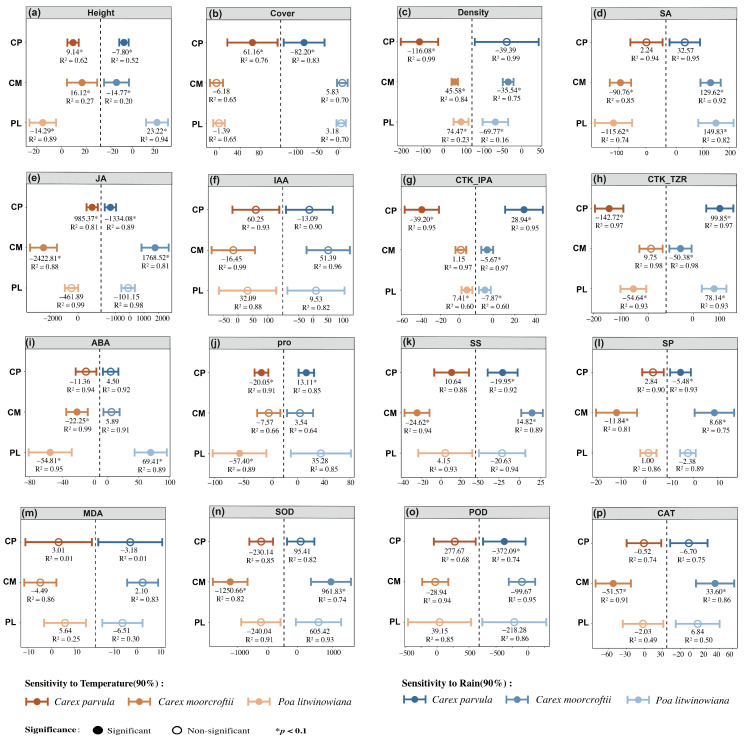
Differential sensitivities of alpine species’ structural and physiological traits to temperature and precipitation changes. Sensitivity was estimated from linear models for each trait under standardized temperature (brown) and precipitation (blue) changes. Filled symbols indicate significance (*p* < 0.05), and R^2^ values denote model fit quality. Species abbreviations: CP (*Carex parvula*), CM (*Carex moorcroftii*), and PL (*Poa litwinowiana*). (**a**–**p**) Height, Cover, Density, SA, JA, IAA, CTK_IPA, CTK_TZR, ABA, Pro, SS, SP, MDA, SOD, POD, and CAT, respectively.

To examine how temperature and precipitation gradients are associated with plant physiological regulation, we applied generalized linear models (GLMs) to calculate standardized response slopes for 15 physiological and structural traits in relation to these meteorological variables (Figure 4). Overall, plant physiological responses were more strongly driven by temperature, as indicated by a greater number of temperature-sensitive traits compared to those related to precipitation. In terms of structural traits, *Poa litwinowiana* showed significant positive responses in height, cover, and density, while *Carex parvula* and *Carex moorcroftii* exhibited mostly negative responses with lower statistical significance. Hormonal traits also differed among species: *Poa litwinowiana* showed positive slopes for IAA, ABA, and TZR, whereas *Carex parvula* and *Carex moorcroftii* showed negative slopes for ABA, IPA, and JA, which are typically linked to stress-related physiological regulation.

Among all physiological indicators, SA and POD were more responsive to precipitation variables in all three species, suggesting that they play a key role in precipitation-driven drought signaling pathways. In contrast, osmoregulation and antioxidant-related indices such as PRO, SOD, and SS were mainly temperature-driven in most of the species, especially in *Carex parvula* and *Carex moorcroftii*, which showed a significant negative response.

Overall, temperature exhibited stronger statistical associations with a larger number of physiological traits across the three species, particularly those related to hormonal balance and osmoregulatory processes, whereas precipitation showed stronger associations with a smaller subset of indicators (e.g., ABA, SA, POD) linked to moisture-related physiological regulation. These patterns highlight differentiated associations of temperature and precipitation with plant physiological traits, and the magnitude and direction of these associations varied among species.

### 2.4. Species-Specific Patterns of Physiological Regulation Across Environmental Gradients

**Figure 5:**
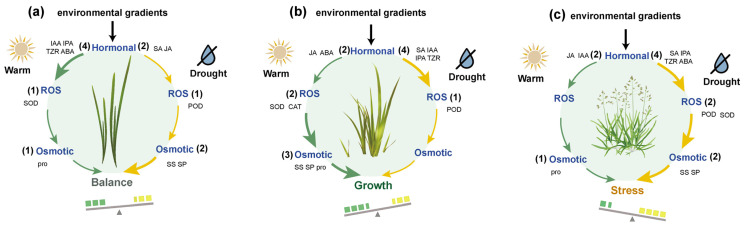
The schematic highlights differences in physiological regulation related to growth-associated and stress-related traits across environmental gradients. (**a**) *Carex parvula*, (**b**) *Carex moorcroftii*, and (**c**) *Poa litwinowiana*. Each species exhibits distinct regulatory patterns along the stress–growth trade-off continuum under combined warming and drought conditions. The diagram highlights three major physiological pathways: ROS scavenging, osmotic adjustment, and hormonal signaling. Within each pathway, the numbers in parentheses (e.g., (1), (2)) represent the number of response indicators (e.g., proline, soluble sugars, specific hormones, antioxidant enzymes) whose variation is predominantly explained by temperature (green arrows) or precipitation (yellow arrows), based on sensitivity analysis. Arrows represent directional responses to environmental drivers, with green pathways primarily induced by warming and yellow pathways by drought.

To further synthesize species-specific physiological response patterns, we constructed an integrative framework based on three major regulatory pathways: hormone regulation, reactive oxygen species scavenging and osmoregulation (Figure 5). This framework illustrates differences in physiological sensitivity patterns and regulatory emphasis among the three alpine species along temperature and precipitation gradients.

Among the three species, *Carex parvula* (Figure 5a) showed a relatively balanced distribution across hormonal regulation, ROS detoxification, and osmotic adjustment pathways, with a similar number of traits primarily influenced by temperature and precipitation. This suggests a stable, well-regulated adaptive pattern. *Carex moorcroftii* (Figure 5b) showed stronger temperature-driven responses in osmotic adjustment traits and maintained relatively stable growth under ROS regulation. In contrast, *Poa litwinowiana* (Figure 5c) was highly sensitive to precipitation, with most hormonal, ROS-related, and osmotic traits predominantly influenced by drought, indicating a stress-prioritized defensive strategy.

This integrative framework highlights species-specific differences in physiological regulation across multiple pathways under contrasting temperature and precipitation conditions. Such differences in physiological sensitivity and regulatory emphasis may contribute to variation in plant growth performance and dominance patterns along environmental gradients, while their broader implications for community composition warrant further investigation.

## 3. Discussion

Overall, our results demonstrate how cold and drying gradients on the Tibetan Plateau shape plant physiological regulation and interspecific differences in performance, providing a mechanistic context for understanding variation in growth-related and dominance-related traits along environmental gradients.

### 3.1. Temperature-Dominated Stability-Driven Versus Precipitation-Dominated Stress Response Pathways

To clarify how different climatic drivers structure physiological regulation, we first examine the relative roles of temperature and precipitation in shaping plant physiological pathways. The GLM results suggest that temperature, as a more stable, long-term climatic factor, exerts more consistent influences across plant physiological processes. Precipitation changes, on the other hand, are more likely to affect pathways highly relevant to drought stress, especially components involved in signal activation and oxidative defense. Temperature-dominated metrics mainly include PRO, IAA and SOD, which are associated with cellular osmoregulation and growth regulation, e.g., IAA acts to promote cell division and elongation, which directly drives bud sprouting and leaf unfolding [7]; precipitation-dominated responses are focused on metrics such as SA and POD, which are commonly associated with stress signaling responses and cellular protection mechanisms [41,42], suggesting that plants are more dependent on defense responses under precipitation variability. Among them, SA is widely recognized as a key regulator in plant immune response. When plants face water stress, they may enhance resistance by increasing SA synthesis, especially in the context of reduced precipitation in the region, and the increase in SA helps to activate the plant’s disease resistance defense system against pathogens and other biotic stresses [43].

### 3.2. Non-Classical Hormonal Response Patterns (ABA–TZR Positive Correlation)

Interestingly, unlike the classic antagonistic relationship between abscisic acid and trans-zeatin riboside, which is commonly interpreted as a trade-off between growth promotion and stress defense [44], our study observed a significant positive correlation between these two hormones across all three species examined (Figure 3). This pattern indicates that, under warming and drying conditions, plants may simultaneously experience signals associated with both growth promotion and water-related stress.

In *Poa litwinowiana*, both ABA and CTK_TZR concentrations increased along the warming and drying gradient. This concurrent increase was accompanied by elevated levels of other stress-related indices, including SA, POD, and SS [45,46]. Together, these patterns suggest a coordinated hormonal response in which growth-related and stress-related signals are both upregulated at the physiological level. Rather than implying a direct regulatory mechanism, this association may reflect a composite physiological state in which plants balance stress responsiveness with the maintenance of growth-related processes. Such a response pattern may enhance drought tolerance and physiological stability at the individual level, potentially supporting greater plant size or height. Although direct evidence for ABA and cytokinin co-regulation under drought conditions remains limited, analogous interactions have been reported between ABA and other hormonal pathways, such as ethylene, in which multiple hormones jointly modulate growth restraint and resource-conserving metabolic states under prolonged stress [47]. In contrast, *Carex parvula* and *Carex moorcroftii* showed relatively stable hormonal responses across the gradient, coupled with stronger clonal expansion and more stable population attributes. This pattern suggests a more conservative physiological response mode that supports population persistence under warming and drying conditions, without reliance on strong hormonal activation or rapid growth responses [39,48].

### 3.3. Interspecific Differences and Response Strategies

In regions such as the Tibetan Plateau, where warming and drying trends are pronounced, interspecific differences in environmental sensitivity may reflect divergent ecological strategies and long-term metabolic tuning to environmental drivers [49,50]. In general, stronger temperature sensitivity may be associated with opportunistic growth responses when thermal conditions are favorable, whereas the activation of precipitation-sensitive pathways may indicate the capacity to cope with short-term drought shocks. These contrasting responses highlight how species prioritize resource allocation among competing physiological functions and, ultimately, how such priorities are expressed as different ecological strategies along environmental gradients.

From a strategy-theory perspective, the two *Carex* species show features consistent with conservative “stress-tolerator” strategies, in which persistence under resource limitation is supported by physiological stability and resource conservation [51]. In particular, *Carex parvula* exhibited relatively stable responses across climatic drivers and pathways, suggesting a wide “environmental tolerance window” and low adjustment costs. Such tolerance-based regulation is likely advantageous under sustained warming–drying conditions, where long-term performance depends less on rapid physiological activation and more on maintaining functional continuity. In contrast, *Poa litwinowiana* displayed broader activation of precipitation-linked stress-response pathways, consistent with a more reactive, stress-responsive strategy that prioritizes defense and short-term buffering but may constrain population establishment when stress persists.

Mechanistically, these physiological strategies may translate into competitive advantages or disadvantages through several pathways. First, allocation trade-offs can determine whether resources are invested in vertical growth and light capture, belowground acquisition, or defense and maintenance. Second, adjustment costs associated with frequent physiological activation may reduce carbon available for clonal spread and recruitment, leading to lower cover and density even when stress responses are strong [52]. Third, life-history traits and clonal architecture can modulate how physiology scales to population dynamics: species with robust tussock or clonal integration may stabilize performance and maintain dominance across microclimatic heterogeneity [53], whereas species relying on rapid metabolic flexibility may show higher responsiveness but also greater demographic volatility.

The divergence in physiological regulatory mechanisms not only influences individual growth performance, but also shapes species’ competitive abilities, resource use efficiency, and environmental adaptability windows—ultimately driving shifts in community structure [54]. When plant species exhibit markedly different sensitivities to meteorological variables, their response timing, adjustment costs, and population establishment will also vary [55]. Such asymmetrical responses can ultimately influence individual survival and drive community-level restructuring by altering the pace of population establishment, potentially leading to species turnover, niche shifts, and localized increases or declines in diversity [56]. This chain of interaction—climate sensitivity, functional construction, and niche patterning—may be key to understanding how alpine meadow biodiversity is being reshaped under warming and drying scenarios. It is also worth noting that these physiological differences may not stem solely from direct responses to climate factors. They are likely constrained by species’ life history traits, root system architecture, and the stability of key functional traits [57,58]. For instance, the relatively stable responses observed in *Carex parvula* may be attributed to its robust root-clump architecture, which helps maintain consistent functionality across diverse microclimatic conditions. Conversely, the high reactivity of *Poa litwinowiana* may reflect a more flexible metabolic system and efficient water-use strategy, enabling rapid adjustment to environmental fluctuations.

### 3.4. Research Limitations and Future Perspectives

Naturally, this study has several limitations. First, the physiological data were collected at a single time point, lacking interannual variation and dynamic response analyses. Accordingly, the present study was designed as a spatial comparison along an elevational gradient, rather than an investigation of temporal dynamics or long-term responses. Second, root functional traits and belowground microbial interactions were not included, which may have led to an underestimation of the role of belowground processes in climate responses. Lastly, the number of sampled species was limited and did not cover a wide range of functional groups or life forms.

Future research should incorporate time-series monitoring and whole-plant trait integration, while also exploring the coordinated responses between aboveground and belowground physiological processes. Such efforts will contribute to the development of a generalized, cross-scale framework for predicting plant physiological responses to environmental gradients. This approach is not only valuable for advancing basic ecological understanding but also has practical implications for ecosystem management and climate-resilient species selection in degraded environments.

## 4. Materials and Methods

### 4.1. Study Area and Sample Selection

**Figure 6:**
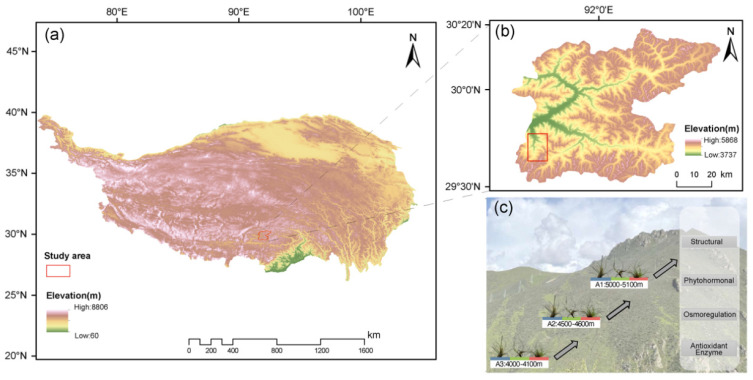
Schematic diagram of the study area and sample collection. (**a**,**b**) Geographic location and elevation distribution of the study area on the Tibetan Plateau. (**c**) Schematic diagram of altitudinal sampling sites (A1–A3) and associated plant physiological measurements.

This study was conducted in a typical alpine region located at the transition zone between the Gangdise and Nyainqentanglha mountain ranges on the Tibetan Plateau (Figure 6). Three distinct elevation zones were selected in this region for plant community analysis and sample collection: A1 (5000–5100 m), A2 (4500–4600 m), and A3 (4000–4100 m). Study plots were selected in areas with minimal human disturbance, ensuring that alpine meadow communities reflected natural vegetation conditions. The experimental plots were located within a fenced alpine meadow area, which effectively excluded grazing by large herbivores and minimized animal disturbance. The study area is characterized by alpine meadow vegetation dominated by herbaceous and shrub species, with no tree canopy present (Table A1). In addition, all plots were established on slopes with the same aspect, thereby minimizing differences in solar radiation and ensuring comparable microclimatic conditions across elevation gradients. Geographic coordinates and elevation were recorded using handheld GPS devices at each plot. Environmental factors such as temperature, air pressure, humidity, and wind speed were continuously monitored by automatic weather stations deployed at different elevation gradients (Table A2). At each elevation gradient, we established 1 m × 1 m sample plots for herbaceous plant community surveys. Five replicate plots were set up at each gradient to enhance data representativeness and stability. Within each plot, we recorded species composition, plant height, cover, density, and leaf thickness of all plant species. For herbaceous species, plant height was defined as the vertical distance from the soil surface to the highest photosynthetic tissue under natural standing conditions, rather than leaf length or stem length. Surveys were conducted during the peak of the growing season to ensure that the data reflected the typical state of the vegetation. Coverage was estimated visually, density was determined by counting the number of individuals per unit area, and height and leaf thickness were measured using a ruler and Vernier caliper. Additionally, 200 g of soil samples were collected from each plot for analysis of soil texture, pH, and available potassium and phosphorus content.

### 4.2. Species Survey, Sample Collection, and Physiological Trait Measurement

The study subjects were three alpine plant species: *Carex parvula* (*Cyperaceae*), *Carex moorcroftii* (*Cyperaceae*), and *Poa litwinowiana* (*Poaceae*). These species are widely distributed in alpine meadows and grasslands of the Tibetan Plateau and are dominant in the study area, exhibiting strong ecological adaptability. In August 2023, during the peak of the growing season, mature leaves of each species were collected from sites at different elevations. Sampling at all elevational sites was conducted within the same time period to ensure comparability. To minimize potential diurnal effects on physiological measurements, sampling at all elevational sites was conducted simultaneously within a fixed time window (09:30–10:30 local time). For each species and elevation, three biological replicates were collected and placed in centrifuge tubes. All samples were immediately wrapped in aluminum foil, rapidly frozen in liquid nitrogen, and stored at −80 °C in an ultra-low temperature freezer prior to physiological and biochemical analyses.

Leaf protein content was determined using the Bradford method [59,60], measured spectrophotometrically at 595 nm. Soluble sugar content was measured using the anthrone colorimetric method proposed by Dubois [61], with absorbance recorded at 620 nm; Proline content was determined using the acidic indophenol colorimetric method [62], with absorbance measured at 520 nm; Malondialdehyde (MDA) content was measured using the thiobarbituric acid (TBA) method [63], with absorbance recorded at 532 nm; Catalase (CAT) activity determination was measured using the UV spectrophotometric method by monitoring the decrease in absorbance at 240 nm [64]. Superoxide dismutase (SOD) activity was measured using the nitroblue tetrazolium method at 560 nm [65], and POD enzyme activity was measured using the guaiacol method at 470 nm [66]. Endogenous plant hormones, including indole-3-acetic acid (IAA), abscisic acid (ABA), trans-zeatin riboside (TZR), jasmonic acid (JA), and salicylic acid (SA), were extracted from leaf tissues following the method of Kettner and Doerffling [67], with minor modifications. Hormone concentrations were determined using liquid chromatography–tandem mass spectrometry (LC–MS/MS) according to established and widely used protocols validated for plant tissues [68], with quantification based on calibration curves using appropriate standards.

### 4.3. Statistical Analysis

First, within each plot, the Importance Value (IV) of each plant species was calculated to reflect its overall dominance in the community. IV was determined using the following formula:

(1) IV = (RH + RC + RD)/3

Here, RH, RC, and RD represent the ratios of a species’ height, cover, and density to the maximum value of each respective metric among all species in the plot, i.e., standardized values.

All physiological data were initially compiled using Microsoft Excel, and subsequent statistical analyses were conducted in R software (version 4.3.3). To investigate the dissimilarity in plant samples across species and elevation gradients, Non-metric Multidimensional Scaling (NMDS) was employed for ordination and visualization. To test whether differences in community composition across species and elevation were statistically significant, Analysis of Similarities (ANOSIM) was conducted. Both NMDS and ANOSIM were performed using the “vegan” package in R [69]. Considering the robustness of one-way analysis of variance (ANOVA) for comparing multiple group means, we applied ANOVA to assess differences in physiological traits among the three species across elevation gradients. Elevation was used as the independent variable, while physiological parameters (e.g., hormone concentrations, osmolytes, and enzyme activities) served as dependent variables. When ANOVA results indicated significant differences (*p* < 0.05), post hoc multiple comparisons were conducted using the Least Significant Difference (LSD) test to identify specific differences between elevation levels. The analyses were implemented using the aov() and LSD.test() functions in the “agricolae” package [70].

To explore the response patterns of physiological indicators to temperature and drought (precipitation) factors and to identify the dominant climatic drivers, we constructed Generalized Linear Models (GLMs) with temperature and precipitation as independent variables and physiological traits (e.g., hormone levels, osmolytes, and antioxidant enzyme activities) as dependent variables. Furthermore, to assess whether the response slopes differed significantly among species for a given physiological indicator, Analysis of Covariance (ANCOVA) was conducted to test interspecific differences in response intensity. The basic form of the GLM used was:

(2) $$\;\;\;\;\;\;Y_{i}=\beta_{0}+\beta_{1}T_{i}+\beta_{2}P_{i}+\epsilon_{i}\;\;\;\;\;\;\;$$

where $Y_{i}\;\;$ represents the physiological trait value of a plant in the i plot, $T_{i}\;$ and $P_{i}$ denote the corresponding temperature and precipitation, $\beta_{0}$ is the intercept, $\beta_{1}$ and $\beta_{2}$ are regression coefficients, and $\epsilon_{i}$ is the error term.

Prior to model fitting, temperature and precipitation were standardized (z-scored) to place predictors on a comparable scale and to facilitate interpretation of relative sensitivity within the same model. Residual normality was assessed using Q–Q plots, and homoscedasticity was evaluated by inspection of residuals versus fitted values.

Except for conceptual diagrams, which were created using Adobe Illustrator (version 2025), all visualizations were generated using the ggplot2 package in R [71], which provides a reproducible framework for data visualization. *p*-values and F or t statistics based on model degrees of freedom are reported in the Appendix A.

## 5. Conclusions

This study demonstrates that alpine plant species exhibit pronounced interspecific differences in physiological regulation along temperature and precipitation gradients. By integrating multidimensional physiological traits with environmental sensitivity analyses, we show that variation in hormonal regulation, osmoregulatory processes, and antioxidant defense pathways is closely associated with differences in plant growth performance and dominance-related structural traits among three dominant alpine species. Temperature was associated with variation in a broader range of physiological traits, particularly those related to growth regulation and osmoregulatory processes, whereas precipitation showed stronger associations with a smaller subset of moisture-related physiological indicators.

These species-specific patterns of physiological regulation highlight contrasting modes of resource allocation and sensitivity to environmental variability among dominant alpine plants. Our findings underscore the importance of considering interspecific differences in physiological responses when interpreting vegetation patterns along environmental gradients. Although the broader implications of these physiological differences for long-term community dynamics remain to be further explored, this study provides a physiological basis for understanding how dominant alpine species respond differently to environmental heterogeneity.

## Figures and Tables

**Table 1:** Community structural traits (mean ± SD) of three dominant alpine species across altitudinal gradients. Traits include plant height, canopy cover, individual density, and importance value (IV), summarized as mean ± standard deviation for each species and elevation level.

Species	Altitude	Height (cm)	Cover (%)	Density (/m^2^)	IV (%)
*Carex parvula*	A1	2.50 ± 0.26 b	74.00 ± 3.61 a	597.00 ± 19.16 a	0.571 ± 0.025 a
	A2	5.83 ± 0.15 a	52.00 ± 2.64 b	362.00 ± 16.37 b	0.394 ± 0.013 b
	A3	4.47 ± 0.55 a	24.67 ± 7.50 c	195.33 ± 7.02 c	0.272 ± 0.027 c
*Carex moorcroftii*	A1	10.43 ± 2.32 a	8.00 ± 1.10 a	35.00 ± 2.65 b	0.208 ± 0.023 b
	A2	13.50 ± 0.95 a	7.80 ± 1.91 a	56.00 ± 6.25 a	0.367 ± 0.036 a
	A3	7.46 ± 0.67 b	8.93 ± 0.51 a	59.33 ± 3.21 a	0.187 ± 0.016 c
*Poa litwinowiana*	A1	28.63 ± 1.40 a	2.00 ± 0.00 b	5.33 ± 1.53 c	0.155 ± 0.01 c
	A2	16.83 ± 2.21 b	4.30 ± 0.46 a	26.33 ± 4.04 a	0.274 ± 0.033 b
	A3	7.50 ± 0.56 c	6.67 ± 2.52 a	15.33 ± 3.78 b	0.326 ± 0.018 a

Values are mean ± SE. Different lowercase letters within the same species indicate significant differences among altitudes (one-way ANOVA followed by LSD test, *p* < 0.05).

**Figure A1:**
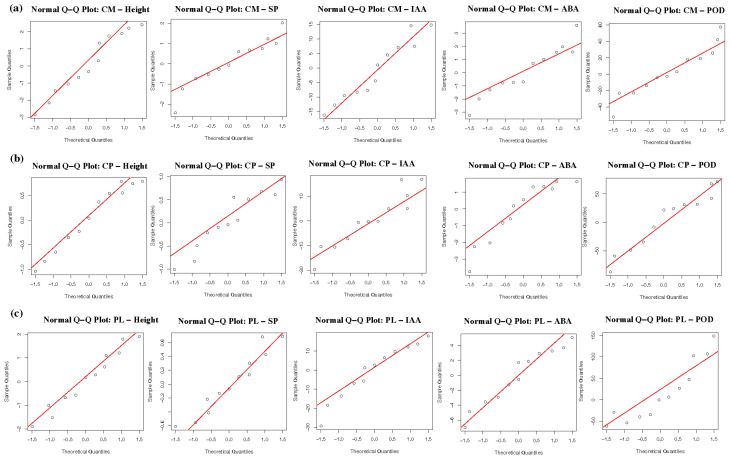
Normal Q–Q plots of residuals from generalized linear models for representative physiological traits across three alpine plant species. (**a**) *Carex moorcroftii* (CM); (**b**) *Carex parvula* (CP); (**c**) *Poa litwinowiana* (PL). Open circles represent ordered residuals (sample quantiles), and the red line indicates the theoretical normal reference line (expected quantiles under normality).

## Data Availability

The raw data supporting the conclusions of this article will be made available by the authors on request.

## Abbreviations

SOC	Soil Organic Carbon
TN	Total Nitrogen
AN	Ammonium Nitrogen
TP	Total Phosphorus
AP	Available Phosphorus
CAT	Catalase
IAA	Indole-3-acetic acid
POD	Peroxidase
SOD	superoxide dismutase
NMDS	Non-metric Multidimensional Scaling
RDA	Redundancy Analysis
SP	Soluble Proteins
MAT	Mean Annual Temperature
RH	Annual Mean Relative Humidity
AMP	Annual Mean Pressure
MAP	Mean Annual Precipitation
WS	Annual Mean Wind Speed
IPA	Isopentenyladenine

## Appendix A

**Table A1:** Structural characteristics of representative alpine plant communities along an elevational gradient. Plots were surveyed at three elevations: A1 (5000 m), A2 (4500 m), and A3 (4000 m), with five replicates per elevation.

Plot	Richness	Total Coverage	Species	Average Height	Sub-Coverage	Density
A1	18	99.3	*Phlomoides rotata*	0.5	3.8	10
			*Leontopodium pusillum*	1.38	0.3	2
			*Carex deasyi*	13.98	4	2
			*Saussurea hieracioides*	3.5	0.3	1
			*Potentilla saundersiana*	1.92	1.2	7
			*Gentiana lhassica*	2.96	6	10
			*Carex moorcroftii*	7.46	8	17
			*Pedicularis alaschanica*	2.46	0.7	9
			*Geranium pylzowianum*	2.75	0.3	2
			*Tripogon bromoides*	7.7	1.8	16
			*Carex parvula*	2.48	74	560
			*Oxytropis glacialis*	2.14	1.5	15
			*Oxytropis microphylla*	3.06	1	7
			*Arenaria bryophylla*	0.38	2.8	3.3
			*Gentiana farreri*	2.65	0.5	2
			*Thalictrum alpinum*	2.62	0.2	21
			*Aster souliei*	7.2	0.1	2
			*Poa litwinowiana*	7.5	2	5
A2	21	94.8	*Salix sclerophylla*	57.00	2.3	1
			*Phlomoides rotata*	2.02	4	12
			*Saussurea hieracioides*	1.13	0.5	3
			*Taraxacum leucanthum*	10.60	0.2	2
			*Geranium pylzowianum*	8.76	1.5	7
			*Potentilla saundersiana*	2.66	2.8	95
			*Polygonum viviparum*	11.98	2.9	150
			*Ranunculus longicaulis*	6.62	2	45
			*Poa litwinowiana*	16.18	4.3	26.25
			*Carex moorcroftii*	13.5	7.8	56.4
			*Carex parvula*	5.82	52	350
			*Carex deasyi*	18.00	9	4
			*Oxytropis microphylla*	5.20	1	37
			*Leontopodium souliei*	5.16	0.5	9
			*Stracheya tibetica*	2.70	0.3	15
			*Pedicularis alaschanica*	6.38	0.8	5
			*Taraxacum sherriffii*	6.27	0.3	3
			*Potentilla anserina*	2.00	0.1	1
			*Aster souliei*	2.75	0.1	2
			*Elymus nutans*	44.98	1	35
			*Ligularia rumicifolia*	63.00	0.5	1
A3	34	73.9	*Potentilla saundersiana*	6.88	1.8	10
			*Cotoneaster microphyllus*	28.16	5.5	5
			*Polygonum tortuosum*	32.00	2	1
			*Pterocephalus hookeri*	1.80	0.3	3
			*Stracheya tibetica*	3.96	2.3	20
			*Phlomis younghushandii*	12.10	1.3	11
			*Polygonum viviparum*	23.42	4	38
			*Youngia simulatrix*	0.62	0.3	20
			*Astragalus englerianus*	8.32	1.1	9
			*Incarvillea younghusbandii*	6.20	1	4
			*Artemisia sieversiana*	7.55	0.2	2
			*Arenaria capillaris* Poiret. var. glandulosa Fenz	6.00	0.1	1
			*Trachydium verrucosum*	5.00	0.1	2
			*Androsace graminifolia*	2.62	0.2	2
			*Thalictrum alpinum*	5.00	0.1	3
			*Polygonum sibiricum*	0.76	2	15
			*Poa litwinowiana*	28.63	6.6	5
			*Dracocephalum tanguticum*	8.45	0.1	2
			*AstragalustribulifoliusBenth.ex Bge.*	2.80	0.2	2
			*Carex deasyi*	15.68	0.1	1
			*Carex parvula*	4.46	24.6	200
			*Stipa capillacea*	36.40	0.5	14
			*Carex moorcroftii*	10.43	9	60
			*Lasiocaryum munroi*	2.58	0.2	4
			*Hymenidium hedinii*	2.40	1	2
			*Stellera chamaejasme* L.	10.88	0.1	4
			*Leibnitzia nepalensis*	1.30	4.3	3
			*Oxytropis microphylla*	6.70	0.5	1
			*Anaphalis xylorhiza*	3.80	0.1	6
			*Leontopodium pusillum*	1.14	0.1	2
			*Saussurea katochaete*	1.20	0.1	1
			*Pedicularis torta*	4.25	0.1	2
			*Pennisetum flaccidum Griseb.*	50.00	0.1	1
			*Elymus nutans*	43.14	3	18

**Table A2:** Elevation, MAT, RH, AMP, MAP, and WS for different sample sites, based on data from a single weather station at each elevation gradient between 2021 and 2023.

Slopes	Elevation (m)	MAT (°C)	RH (%)	MAP (mm)	WS (m/s)	Meteorological Station Location/Elevation (m)
A3	5000–5100	0.6	16.3	919.9	9.2	Phase II TSF/5060
A2	4500–4600	1.7	14.9	757.6	10.7	TBM Launching Portal/4750
A1	4000–4100	3	35.3	598.3	7.1	TSF/4140
